# A zone-of-inhibition assay to screen for humoral antimicrobial activity in mosquito hemolymph

**DOI:** 10.3389/fcimb.2023.891577

**Published:** 2023-01-26

**Authors:** Bianca Morejon, Kristin Michel

**Affiliations:** Division of Biology, Kansas State University, Manhattan, KS, United States

**Keywords:** innate immunity, mosquito, toll pathway, antimicrobial peptide (AMP), defensin 1, ZOI (zone of inhibition)

## Abstract

In insects, antibacterial immunity largely depends on the activation of downstream signaling and effector responses, leading to the synthesis and secretion of soluble effector molecules, such as antimicrobial peptides (AMPs). AMPs are acute infection response peptides secreted into the hemolymph upon bacterial stimulation. The transcription of innate immunity genes encoding for AMPs is highly dependent on several signaling cascade pathways, such as the Toll pathway. In the African malaria mosquito, *Anopheles gambiae*, AMPs hold a special interest as their upregulation have been shown to limit the growth of malaria parasites, bacteria, and fungi. Most of the current knowledge on the regulation of insect AMPs in microbial infection have been obtained from *Drosophila*. However, largely due to the lack of convenient assays, the regulation of antimicrobial activity in mosquito hemolymph is still not completely understood. In this study, we report a zone of inhibition assay to identify the contribution of AMPs and components of the Toll pathway to the antimicrobial activity of *A. gambiae* hemolymph. As a proof of principle, we demonstrate that *Micrococcus luteus* challenge induces antimicrobial activity in the adult female mosquito hemolymph, which is largely dependent on defensin 1. Moreover, by using RNAi to silence *Cactus*, *REL1*, and *MyD88*, we showed that *Cactus* kd induces antimicrobial activity in the mosquito hemolymph, whereas the antimicrobial activity in *REL1* kd and *MyD88* kd is reduced after challenge. Finally, while injection itself is not sufficient to induce antimicrobial activity, our results show that it primes the response to bacterial challenge. Our study provides information that increases our knowledge of the regulation of antimicrobial activity in response to microbial infections in mosquitoes. Furthermore, this assay represents an *ex vivo* medium throughput assay that can be used to determine the upstream regulatory elements of antimicrobial activity in *A. gambiae* hemolymph.

## Introduction

Mosquitoes vector the causative agents of several infectious diseases worldwide (Molina-Cruz et al., 2016; Ramasamy and Surendran, 2016; Burkett-Cadena and Vittor, 2018). The *Anopheles* mosquito species mainly transmits *Plasmodium* parasites, the causative agents of malaria, one of the most widespread and devastating parasitic infections in humans (Molina-Cruz et al., 2016). In the absence of sterilizing vaccines, malaria prevention relies largely on vector control using insecticides and bed nets and, to a lesser extent, on drug treatment of most at risk populations (Lengeler, 1998; Killeen et al., 2007; Nosten and White, 2007). However, since the 1950s, insecticide resistance in mosquitoes has been reported towards different classes of insecticides, including pyrethroids, organophosphates, and carbamates (Hemingway et al., 2002; Goindin et al., 2017). Thus, new vector control strategies must be designed and implemented (Kleinschmidt et al., 2018). The mosquito immune system is a potential novel target for such alternative measures, as the immune response initiated in these insects during infection with vector-borne disease agents is a key determinant of vector competence (Huff, 1927; Collins et al., 1986; Vernick et al., 1995; Blandin et al., 2004; Osta et al., 2004; Michel et al., 2005; Habtewold et al., 2008; Edgerton et al., 2020).

To face the threat of infection due to the recurrent and diverse microbial exposure throughout their life cycle, mosquitoes deploy their innate immune system encompassing a variety of synergistic defense mechanisms (Levashina, 2004; Yassine et al., 2012; Tikhe and Dimopoulos, 2021). Binding of recognition molecules to microbe-specific molecules activates two principal arms of immune response: the humoral and cellular components. This immune response is mediated by factors that act as regulators and effectors of cellular responses—such as phagocytosis and encapsulation—and humoral responses—such as melanization and coagulation—among others (Lai et al., 2001; Lai et al., 2002; Dimopoulos, 2003; Levashina, 2004; Meister et al., 2005; Nakhleh et al., 2017). To identify and detect factors that contribute to antimicrobial immunity in *A. gambiae*, several assays have been developed, including the observation of phagocytosis *in vivo* (Levashina et al., 2001; Moita et al., 2005; Moita et al., 2006), evaluation of mosquito survival after injection of live bacteria (Blandin et al., 2002; Meister et al., 2005; Molina-Cruz et al., 2008; Gupta et al., 2009; Coggins et al., 2012; Upton et al., 2015), and quantification of bacterial proliferation after challenge (Schnitger et al., 2007; Warr et al., 2008; Schnitger et al., 2009; Yassine et al., 2012). However, these assays have a limited ability to distinguish between factors contributing to either or both arms of the immune response (Dimopoulos et al., 1997; Christophides et al., 2004; Levashina, 2004; Hillyer and Strand, 2014).

Recognition of microbial molecules also activates signaling cascades such as the Toll and Imd pathways, which, within hours after challenge, induce the synthesis of hundreds of immune-inducible molecules, including the well-characterized antimicrobial peptides (AMPs) (Levashina, 2004; Zakovic and Levashina, 2017). Knowledge on the components of the Toll pathway, as well as the interactions between these components in *Drosophila*, has served as a blueprint for studying it in mosquitoes. In Toll pathway signaling in *A. gambiae*, detection of pathogen-derived ligands by pattern recognition receptors (PRRs) triggers the proteolytic cleavage of the cytokine Spätzle, which binds to and activates the Toll receptor. This initiates signaling through the death-domain adaptor proteins MyD88, Tube, and Pelle, resulting in the phosphorylation and degradation of CACTUS, a negative regulator which binds to and inhibits the NF-κB transcription factor REL1 in the cytoplasm (Barillas-Mury et al., 1996; Cramer et al., 1999). CACTUS degradation allows REL1 to translocate into the nucleus to activate the transcription of Toll pathway-regulated genes, such as the AMPs *Cecropin 1 (CEC1*) and *Defensin 1* (*DEF1*) (Frolet et al., 2006; Luna et al., 2006; Garver et al., 2009).

Upon immune induction, newly synthesized AMPs are secreted into the hemolymph where they rapidly reach micromolar concentrations, making them detectable in hemolymph after challenge (Lowenberger et al., 1999; Vizioli et al., 2001b). A variety of microorganisms have been used to induce AMP synthesis in the hemolymph of insect larvae and adults, with *E. coli*, *M. luteus*, *Enterobacter cloaceae*, and *Pseudomonas aeruginosa* being the most commonly used bacterial species (Hoffmann et al., 1981; Qu et al., 1982; Bulet et al., 1991; Sumida et al., 1992; Boulanger et al., 2002; Huberman et al., 2007a; Randolt et al., 2008). Quantification of *AMP* mRNA is commonly used as a measure for immune induction (Dickinson et al., 1988; Richman et al., 1996; Lemaitre et al., 1997; Lowenberger et al., 1999; Christophides et al., 2002; Dai et al., 2008; Zhang et al., 2017) and to test for the activity of the Toll and IMD pathways (Lemaitre et al., 1995; Meng et al., 1999; Meister et al., 2005; Frolet et al., 2006; Antonova et al., 2009; Meister et al., 2009; Zhong et al., 2012; Zhang et al., 2017). However, the relative contribution of signaling pathways that contribute to the expression of individual AMPs is largely unclear in mosquitoes (Meister et al., 2005; Frolet et al., 2006; Luna et al., 2006; Garver et al., 2009). Furthermore, the relationship between AMP mRNA and peptide levels can be complicated as demonstrated for defensin in *A. aegypti* (Bartholomay et al., 2004), and thus AMP mRNA levels may not correlate well with overall antimicrobial activity in mosquito hemolymph. AMPs can be purified instead from hemolymph or produced by recombinant DNA technologies to further study their biochemical properties and antimicrobial activity (Hultmark et al., 1983; Bulet et al., 1991; Richman et al., 1996; Otvos, 2000; Vizioli et al., 2001a; Buonocore et al., 2021). To quantify the antimicrobial activity of AMPs, *in vitro* antimicrobial sensitivity assays are commonly performed by measuring the zone of bacteria growth inhibition produced after immune stimulation in *Antheraea pernyi* (Qu et al., 1982), *Trichoplusia ni* (Andersons et al., 1990), *Bombyx mori* (Sumida et al., 1992), *Stomoxys calcitrans* (Boulanger et al., 2002), *Apis mellifera carnica* (Randolt et al., 2008), and *Lucilia sericata* (Huberman et al., 2007a). This method has allowed the quantification of antimicrobial activity of several AMPs such as cecropins, defensins, and gambicin in *Anopheles gambiae* (Vizioli et al., 2000; Vizioli et al., 2001b), attacins in *Hyalophora cecropia* (Hultmark et al., 1983), as well as other immune factors such as lysozymes in *Gryllus bimaculatus* and *Heliothis virescens* (Schneider, 1985; Lockey and Ourth, 1996).

In this study, we establish an assay that allows the detection of factors contributing specifically to humoral antimicrobial activity in the hemolymph of adult female mosquitoes. This assay is based on zone-of-inhibition measurements using *M. luteus* after bacterial challenge and/or reverse genetic manipulations. We use this assay to determine the contributions of (1) two antimicrobial peptides and (2) the Toll pathway to this antimicrobial activity. In addition, the data presented here show that this assay can also be used to detect immune priming.

## Materials and methods

### Mosquito strain and maintenance

The *A. gambiae* G3 strain (MRA-112) was reared as described previously (An et al., 2011). Briefly, mosquitoes were maintained at 27°C and 80% humidity in an environmental chamber set to 12:12-h light/dark cycle. L1 larvae were fed 2% (w/v) suspension of baker’s yeast (Fleischmann’s Active Dry Yeast, AB Mauri, St. Louis, MO, USA), and L2–L4 instars were fed on a 2:1 mixture of 2% powdered fish food (TetraMin^®^ Tropical Flakes, Tetra, Melle, Germany) and baker’s yeast. Pupae were collected manually and placed in 173-oz plastic cages (CL Smith Co, St. Louis, MO, USA) covered with netting (JoAnn, Hudson, Ohio, USA). After emergence, adult mosquitoes were provided with 8% fructose *ad libitum* and heparinized horse blood (PlasVacc, Templeton, CA, USA) used as a blood meal source provided through an artificial membrane feeding system consisting of glass bells and a circulating water bath (Thermo Fisher Scientific, Waltham, MA, USA).

### Bacterial and fungal strains

The following bacterial strains were used for bacterial challenge and zone of inhibition (ZOI) assays: *Micrococcus luteus* (*lysodeikticus*) (ATCC, no. 4698), live *Staphylococcus aureus* [strain PIG1; Liu et al., 2005)], and *Escherichia coli* (OP50, NCBI:txid637912). ZOI assays were also performed with *Saccharomyces cerevisiae* (baker’s active dried yeast from MP Biomedicals, Solon, OH, USA).

### Bacterial challenges

To prepare the bacterial suspensions for mosquito challenges, lyophilized *M. luteus* (MilliporeSigma, Burlington, MA, USA) was resuspended in 1× phosphate-buffered saline (PBS) to an optical density of 600 nm (OD_600_) = 5. For live bacterial challenges, *E. coli* and *S. aureus* were cultured at 37°C in Luria-Bertani (LB) broth overnight, washed with 1× PBS, and resuspended in 1× PBS at the same optical density as mentioned above. The OD_600_ = 5 value corresponded to average doses of 8.5 × 10^8^ and 9.7 × 10^8^ colony-forming units (CFUs)/ml for live *E. coli* and *S. aureus*, respectively.

In all experiments, 2-day-old adult female mosquitoes were injected under the wing base with 50.6 nl of the bacterial challenge using a nanoinjector (Nanoject III, Drummond Scientific, Broomall, PA, USA). Sterile 1× PBS injections were used as negative controls. The experiments were performed in three independent replicates with 40 mosquitoes per treatment and per replicate.

### Hemolymph collection

Unless otherwise stated, hemolymph was collected 24 h after challenge by adapting the method developed by League et al. (2017). Briefly, the lateral thorax of 40 CO_2_-anesthesized mosquitoes per treatment was punctured using a 30G × ½″ sterile needle. The freshly wounded mosquitoes were placed into a 0.6-ml microfuge tube whose bottom was perforated using an 18G × 1 ½× needle. This tube was then inserted into a 1.5-ml microfuge tube and centrifuged at 5,500 rpm for 5 min at 4°C. The collected hemolymph (~2 µl per treatment) was flash-frozen in liquid nitrogen immediately after collection and then stored at -80°C until use.

### Zone of inhibition assays

To measure the antimicrobial activity of hemolymph, ZOI assays were performed as adapted from the protocol developed by League et al. (2017). Briefly, bacterial cultures were grown overnight in LB broth at 37°C in a shaking incubator to OD_600_ = 10, which corresponds to average doses of 3.5 × 10^10^, 7.4 × 10^10^, and 8.1 × 10^10^ CFUs/ml for *E. coli*, *S. aureus*, and *M. luteus*, respectively. Moreover, 500 ul of the bacterial culture was used to seed 5.5 ml of 1% LB agar kept in a 55°C water bath and then poured onto a 9-cm-diameter Petri dish (Fisher Scientific, Waltham, MA, USA). Cultures and plates using *S. cerevisiae* were prepared as described above, substituting LB for yeast extract–peptone–dextrose. The OD_600_ = 10 value for this yeast species corresponded to average doses of 2.6 × 10^9^ CFUs/ml. After the agar had solidified, equidistant wells (1.5 cm from the outer edge) were punched in the agar by using a 1-mm-tip, sterile, 9″ glass Pasteur pipette (Fisher Scientific, Waltham, MA, USA). Each well was then loaded with 1 µl of hemolymph per treatment. Once the hemolymph was absorbed by the agar, usually within a couple of minutes, the plates were incubated upside down for 16 h at 37°C, unless otherwise stated. The collected hemolymph from each treatment and replicate was tested twice using different batches of bacterial culture; hence, six ZOI trials per treatment group were performed.

### Bacterial plate imaging and ZOI data analysis

The plates were imaged at 16.1 pixels per millimeter using the Azure 300 imaging system (Azure Biosystems Inc., Dublin, CA, USA), using white epi-illumination without filters. Image processing and ZOI measurement were performed in Fiji is Just Image J (Fiji, Version 2.1.0) (Schindelin et al., 2012) using the following macro: “run(“8-bit”); run(“Subtract Background…”, “rolling=100 light”); run(“Auto Threshold”, “method=Default white”); run(“Convert to Mask”); run(“Analyze Particles…”, “size=400-Infinity pixel show=Outlines display”);”. **Supplementary Figure S1** provides an overview of the entire procedure.

### dsRNA synthesis and RNAi in female mosquitoes

dsRNA synthesis and adult female mosquito injections were performed as described previously (An et al., 2011). The templates for dsRNA were prepared by PCR using the T7-tagged primers listed in **Supplementary Table S1**. In all experiments, 2-day-old adult female mosquitoes were injected with 69 nl of 3 µg/µl dsRNA for single-knockdown (kd) experiments. For kd control, the mosquitoes were injected with the same quantity of the non-related dsRNA of green fluorescent protein (*GFP*). To examine the genetic interactions between *REL1*, *MyD88*, and *CACTUS*, a double-knockdown (dkd) was performed by injecting 138 nl of a 1:1 solution of 1.5 µg/ul of each dsRNA. For dkd controls, ds*GFP* was added to keep the total dsRNA dose constant at 414 ng/mosquito between treatment and controls as described previously (Zhang et al., 2016).

In experiments that required immune activation after gene kd, the mosquitoes were challenged 48 h after dsRNA injection with resuspended lyophilized *M. luteus*, live *S. aureus*, and *E. coli*, respectively, as described in the Bacterial Challenge Section Hemolymph was collected 24 h after challenge as described in the previous section.

### Real-time quantitative polymerase chain reaction

To test for gene knockdown efficiency after dsRNA treatment, eight female mosquitoes per treatment and replicate were collected 48 h after dsRNA injection for RT-qPCR. Total RNA extraction and template cDNA synthesis were performed as described previously (Rhodes et al., 2018). Purified total RNA (100 ng) was used as the template to synthesize cDNA using iScript cDNA synthesis kit (Bio-Rad, Hercules, CA, USA) in a total reaction volume of 20 μl, following the manufacturer’s instructions. RT-qPCR was performed using PowerUp SYBR Green Master Mix (Thermo Fisher Scientific, Waltham, MA, USA) according to the manufacturer’s protocol. Briefly, 2 μl of 1:5 diluted cDNA was added as template for a 20-μl volume reaction, followed by amplification on the Applied Biosystems™ 7500 Real-Time PCR System using the following amplification protocol: an initial cycle of 2 min at 50°C followed by 2 min at 95°C, 40 cycles of 15 s at 95°C, and 1 min at 60°C (detection). The primers used are listed in **Supplementary Table S2**.

The relative expression of the genes of interest was calculated according to Pfaffl (2001), considering the primer efficiencies (**Supplementary Table S2**). Ribosomal protein *S7* (*RpS7*) expression was used as reference and ds*GFP* treatments as calibrator conditions. Three technical replicates were measured for each sample and primer pair from three biological replicates.

### Statistical analyses

Statistical analyses were performed using GraphPad Prism 6.07 Software (GraphPad Software, USA). The area of bacteria growth inhibition (mm^2^) data was evaluated for normality of distribution using the Kolmogorov–Smirnov normality test. Differences in the zone of inhibition were analyzed using one-way ANOVA for multiple-treatment groups, with Bonferroni’s multiple-comparisons post-test (*P* < 0.05).

## Results

### Bacterial challenge induces antimicrobial activity in mosquito hemolymph

**Figure 1:**
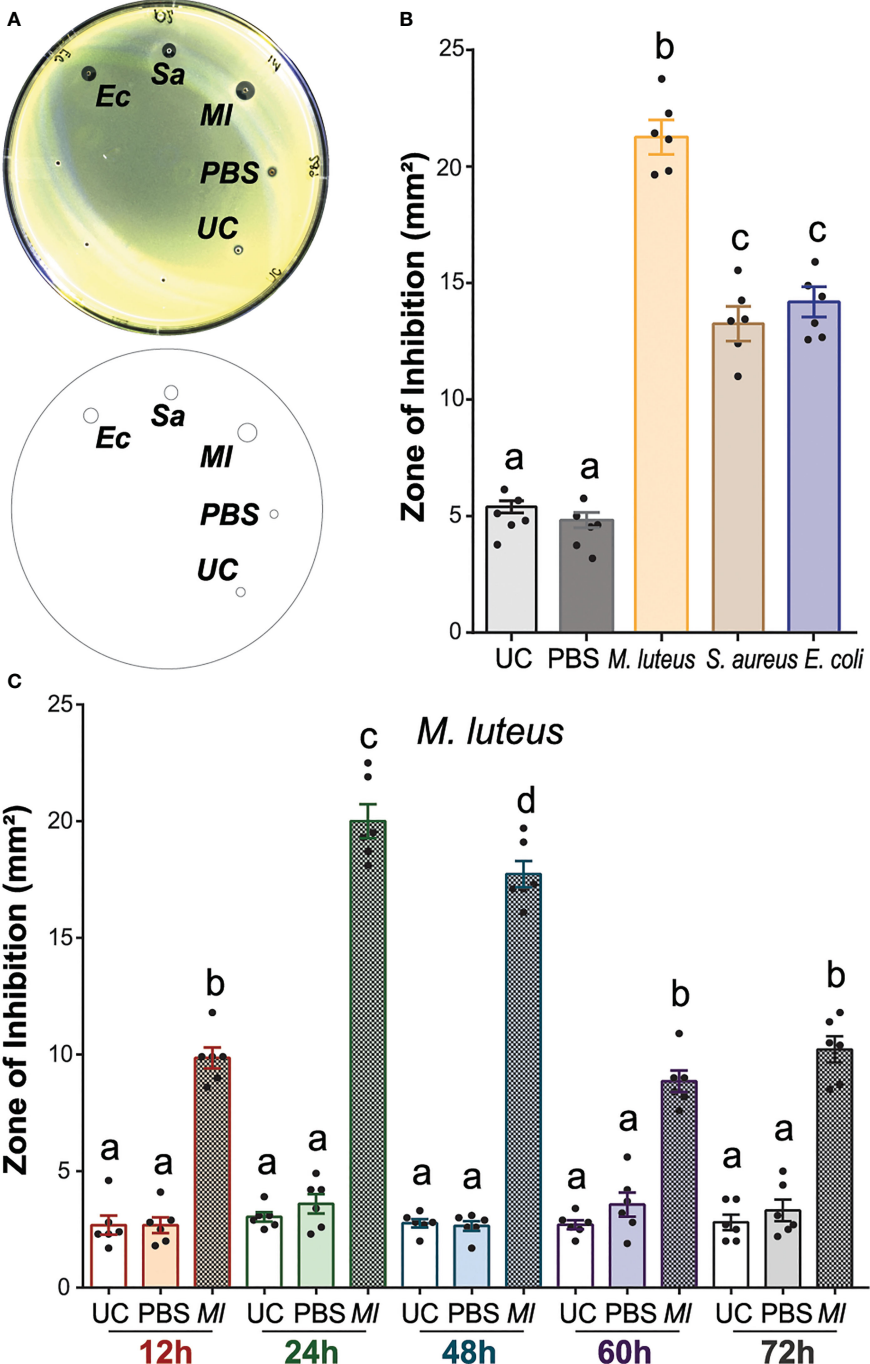
The antimicrobial activity of *Anopheles gambiae* hemolymph is induced by bacterial challenge. **(A)** Top: *M. luteus*-seeded plate showing the zone of inhibition (ZOI) produced by mosquito hemolymph collected 24 h after challenge with *E coli*, *S. aureus*, and *M. luteus*, respectively. The hemolymph of uninjected and phosphate-buffered-saline-injected mosquitoes acted as negative controls. Bottom: Drawing of the plate generated in Fiji is Image J to quantify the zone of inhibition (ZOI) for each treatment. **(B)** Zones of bacterial growth inhibition (mm^2^) were measured using customized macros in Fiji is Image J **(C)** The temporal variation of antimicrobial activity induction with *M. luteus* challenge was determined by collecting hemolymph at 12, 24, 48, 60, and 72 h after challenge. The ZOI was then measured as in **(B)**. In **(B) (C)**, data are shown as means with ±1 standard error (*n* = 6). One-way ANOVA, followed by Bonferroni’s multiple-comparison post-test, was performed to calculate the statistical significance (*P* < 0.05). Means with the same letter are not significantly different.

To establish a method to quantify the antimicrobial activity in *A. gambiae*, we performed ZOI assays in plates seeded with *M. luteus* using the hemolymph of mosquitoes after lyophilized *M. luteus*, live *E. coli*, and *S. aureus* challenges. We selected *M. luteus* for the challenge as it is a well-known inducer of immunity in several insect species (Nasr and Fallon, 2003; Huberman et al., 2007b; Kajla et al., 2010). In addition, we employed *E. coli* and *S. aureus*, which had been used in previous studies of mosquito antimicrobial immunity (Dimopoulos et al., 1997; Blandin et al., 2002; Garcia-Lara et al., 2005; Dong et al., 2006; Eleftherianos et al., 2006; Rahnamaeian et al., 2015). Injection of bacteria did not alter the mosquito mortality within the first 24 h post-injection compared with PBS-injected controls, with one to two mosquitoes dying within this time frame. The antimicrobial activity of hemolymph among all five treatment groups revealed significant differences (**Figures 1A, B**; one-way ANOVA, *df* = 4, *F* = 137.7, *P* < 0.0001). The hemolymph of unchallenged (UC) mosquitoes had low antimicrobial activity, producing a ZOI of 4 to 5 mm^2^. This activity was not increased by PBS injection, as the size of the ZOI did not statistically significantly differ from that of the UC mosquito hemolymph. When mosquitoes were challenged with either bacterial species, the ZOI was 1.9–3.4-fold larger than that of the control hemolymph, indicating a higher antimicrobial activity after challenge. Among the different challenges, the *M. luteus* challenge produced the largest ZOI, with a 1.5- and 1.6-fold increase in size compared with *E. coli* and *S. aureus*, respectively. Overall, the antimicrobial activity in mosquito hemolymph was increased significantly after the bacterial challenge, with *M. luteus* producing a higher antimicrobial activity compared with *E. coli* and *S. aureus* (Bonferroni’s multiple comparisons test, *P* < 0.0001 for both comparisons).

To determine whether the antimicrobial activity of hemolymph could inhibit the growth of other microbial species, we seeded the plates with *M. luteus*, *E. coli*, *S. aureus*, and *S. cerevisiae*, respectively, and tested the hemolymph of UC and *M. luteus*-challenged females. No ZOI was observed in plates seeded with *E. coli*, *S. aureus*, and *S. cerevisiae*, respectively (**Supplementary Figure S2A**). The plates seeded with *M. luteus* showed the same patterns of hemolymph-induced antibacterial activity as described above. To test whether the reduction in bacterial replication rates of *E. coli*, *S. aureus*, and *S. cerevisiae* would allow the detection of antimicrobial activity, we reduced the incubation temperature to 27°C, which is the typical temperature used in *A. gambiae* rearing. No ZOI was detected in either of these plates (**Supplementary Figure S2B**). Finally, we tested whether using the same bacterial species for both, challenging the mosquito and seeding the plates, would show inhibition of bacterial growth. For this, we collected hemolymph from mosquitoes challenged with *E. coli* and *S. aureus*, respectively, and tested those hemolymph samples in plates seeded with their corresponding bacterial species. No ZOI again was observed in either of these plates (**Supplementary Figure S2C**). Therefore, *A. gambiae* hemolymph only inhibited the growth of *M. luteus*, not *E. coli*, *S. aureus*, or *S. cerevisiae*, as measured by the ZOI assays. This was independent of the incubation temperature of the bacterial plates and the microbial challenge used to induce antimicrobial activity in hemolymph.

### Antimicrobial activity in mosquito hemolymph remains elevated for 3 days post-challenge

To determine the longevity of the observed increase in antimicrobial activity in hemolymph, we next analyzed the temporal variation of the immune induction after *M. luteus* challenge, as this species produced the highest antimicrobial activity from the tested bacterial challenges (**Figure 1C**). For this, we collected hemolymph at 12, 24, 48, 60, and 72 h after *M. luteus* challenge and from uninjected mosquitoes (UC). Low antimicrobial activity was observed in all UC hemolymph samples, producing ZOI of 3 to 4 mm^2^ across all timepoints. PBS injection did not increase the antimicrobial activity at any time point. The antimicrobial activity of hemolymph was significantly increased after *M. luteus* challenge compared with UC and PBS-injected controls in every timepoint (one-way ANOVA, *df* = 14, *F* = 180, *P* < 0.0001, Bonferroni’s post-test, *P* < 0.0001). At the 12-h timepoint, *M. luteus* challenge produced a ZOI 2.7-fold larger than the UC. The antimicrobial activity after challenge peaked at 24 h, with a ZOI 5.6-fold larger than the UC and twofold larger than at the 12-h timepoint. The activity at 48 h after *M. luteus* injection was slightly reduced compared with the 24-h timepoint but remained high overall, with a ZOI 5.4-fold larger than the UC at the corresponding timepoint. At 60 and 72 h, the antimicrobial activity reached levels comparable with the 12-h timepoint, producing a ZOI 2.3- and 2.6-fold larger than its corresponding UC, but 55.8% and 50.1% smaller than challenge-induced hemolymph at 24 and 48 h, respectively. Overall, the antimicrobial activity in mosquito hemolymph was significantly induced after *M. luteus* challenge in all the timepoints that we tested. This activity peaked at 24 h and remained high at 48 h, followed by 2× decrease, after which the activity plateaued. These results together establish that changes in antimicrobial activity can be detected in the mosquito hemolymph and quantified using ZOI assays. This antimicrobial activity of hemolymph was induced with different bacterial challenges, of which *M. luteus* challenge induced the highest antimicrobial activity 24 h after bacterial injection.

### Antimicrobial activity against *Micrococcus luteus* largely depends on defensin 1

**Figure 2:**
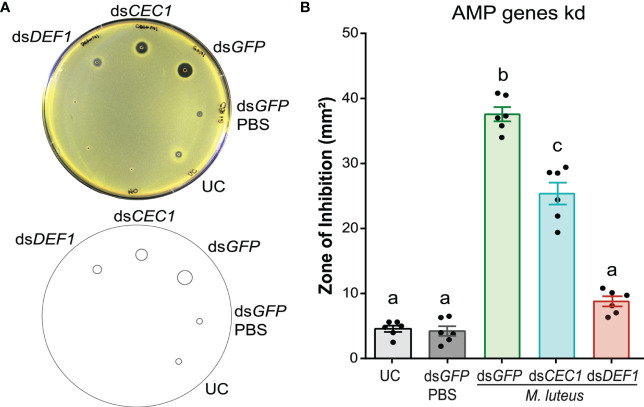
Defensin 1 is mainly responsible for the antimicrobial activity against *M. luteus*. **(A)** Top: *M. luteus*-seeded plate showing the zone of inhibition (ZOI) produced by the hemolymph of ds*DEF1-* and ds*CEC1*-injected mosquitoes. The mosquitoes were challenged 48 h after dsRNA injection with *E coli*, *S. aureus*, and *M. luteus*, respectively. Hemolymph was collected 24 h after challenge. Mosquitoes that were uninjected and injected with ds*GFP* prior to phosphate-buffered saline acted as negative controls. Bottom: Drawing of the plate generated in Fiji is Image J to quantify the ZOI for each treatment. **(B)** Zones of bacterial growth inhibition (mm^2^) were measured using customized macros in Fiji is Image J Data are shown as means with ±1 standard error (*n* = 6). One-way ANOVA, followed by Bonferroni’s multiple-comparison post-test, was performed to calculate the statistical significance (*P* < 0.05). Means with the same letter are not significantly different.

The secretion of AMPs after microbial challenge increases the antimicrobial activity of insect hemolymph (Uvell and Engström, 2007). To test the contribution of AMPs on the antimicrobial activity of mosquito hemolymph, we used RNAi prior to *M. luteus* challenge to silence two known *A. gambiae* AMP genes, *Defensin 1* (*DEF1*) and *Cecropin 1* (*CEC1*), respectively (**Figure 2**). The kd efficiencies of *DEF1* and *CEC1* were measured by RT-qPCR 48 h after dsRNA injections (**Supplementary Table S3**). A comparison of the antimicrobial activity of hemolymph between treatment and control groups revealed significant differences (one-way ANOVA, *df* = 4, *F* = 202.1, *P* < 0.0001). As in the experiments detailed above, low antimicrobial activity was observed with the hemolymph of UC mosquitoes, producing a ZOI of 4 to 5 mm^2^. This activity did not increase in mosquitoes injected with ds*GFP* (dsRNA negative control), followed 48 h later by PBS (bacterial carrier) injection (Bonferroni’s post-test, *P* > 0.999). As expected, antimicrobial activity was highly increased (7.2-fold) when ds*GFP*-injected mosquitoes were challenged with *M. luteus*. ds*DEF1* injection lowered the antimicrobial activity after challenge to levels comparable with the UC. Similarly, ds*CEC1* injection also reduced the antimicrobial activity compared with ds*GFP*-injected, *M. luteus*-challenged mosquitoes. However, the antimicrobial activity after ds*CEC1* injection remained 4.5-fold elevated compared with the UC, suggesting that DEF1 was mainly responsible of the antimicrobial activity against *M. luteus* in the mosquito hemolymph, while CEC1 contributed to a lesser degree.

### The Toll pathway contributes to antimicrobial activity in *A. gambiae* hemolymph

**Figure 3:**
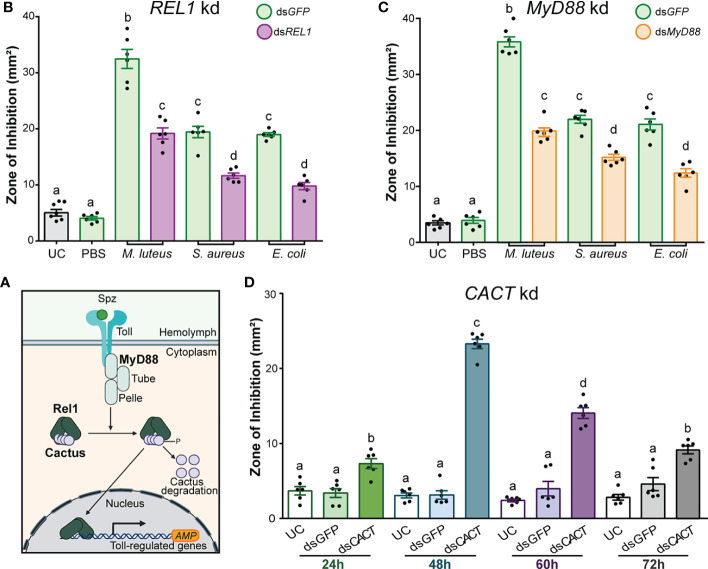
The antimicrobial activity of *Anopheles gambiae* hemolymph is regulated by the Toll pathway. **(A)** Overview of the Toll signal transduction pathway showing the known components that act as positive and negative regulators of the transcription of AMP genes in *Anopheles gambiae*. **(B)** Zone of inhibition produced by the hemolymph of ds*REL1*-injected **(B)** and ds*MyD88*-injected **(C)** mosquitoes. The mosquitoes were challenged 48 h after ds*RNA* injection with *E coli*, *S. aureus*, and *M. luteus*, respectively. Hemolymph was collected 24 h after challenge. Mosquitoes that were uninjected (UC) and injected with ds*GFP* prior to phosphate-buffered saline acted as negative controls, whereas those which had ds*GFP* injection prior to *E coli*, *S. aureus*, and *M. luteus* challenges acted as the corresponding positive controls. **(D)** The temporal variation of antimicrobial activity induction after *CACT* kd was determined by collecting hemolymph at 24, 48, 60, and 72 h after ds*CACT* injection. In **(B)**, **(C)**, and **(D)**, data are shown as means ±1 standard error (*n* = 6). One-way ANOVA, followed by Bonferroni’s multiple-comparison post-test, was performed to calculate the statistical significance (*P* < 0.05). Means with the same letter are not significantly different.

To identify the contribution of the Toll signal transduction pathway to the antimicrobial activity of *A. gambiae* hemolymph, we used RNAi to silence the three components of the pathway: *REL1*, *MyD88*, and *Cactus* (*CACT*) (**Figure 3A**). The kd efficiencies of *REL1*, *CACT*, and *MyD88* were measured by RT-qPCR 48 h after dsRNA injections (**Supplementary Table S3**).

Silencing *REL1* (the NF-κB transcription factor purportedly downstream of TOLL) prior to *M. luteus*, *E. coli*, and *S. aureus* challenge significantly reduced the hemolymph antimicrobial activity compared with ds*GFP-*injected control mosquitoes (**Figure 3B**; one-way ANOVA, *df* = 7, *F* = 187, *P* < 0.0001; Bonferroni’s post-test, *P* < 0.0001 for all challenge comparisons). The bacterial challenge after ds*GFP* injection produced a several-fold increase in ZOI compared with UC hemolymph. Fold induction was highest after the challenge with *M. luteus* (5.5-fold), followed by *S. aureus* (2.9-fold) and *E. coli* (2.8-fold). Injection of ds*REL1* reduced the fold induction of antimicrobial activity by 40%–50%, compared with the ds*GFP*-injected mosquitoes challenged with the same bacterial species. Therefore, the antimicrobial activity in the *A. gambiae* hemolymph was significantly reduced in *REL1*-silenced mosquitoes after the bacterial challenge independent of the microbial species that we tested. While not the focus of this current study, dsRNA injection targeting *REL2*, the transcription factor downstream of the IMD pathway in mosquitoes, reduced the antimicrobial activity in *A. gambiae* hemolymph similarly to *REL1* kd (**Supplementary Figure S3**). The kd efficiency of *REL2* was measured 48 h after dsRNA injections (**Supplementary Table S3**).

A similar effect was observed after silencing *MyD88*, one of the death domain adaptor proteins in the Toll intracellular signaling cascade (**Figures 3A, C**). Silencing *MyD88* prior to *M. luteus*, *E. coli*, and *S. aureus* challenge produced a significantly lower antimicrobial activity than in the ds*GFP-*injected control mosquitoes (**Figure 3C**; one-way ANOVA, *df* = 7, *F* = 216.2, *P* < 0.0001; Bonferroni’s post-test, *P* < 0.0001 for all challenge comparisons). The *M. luteus* challenge after ds*GFP* injection produced a ZOI 9.3-fold larger than the UC, whereas the ZOI after ds*MyD88* injection was only 4.7-fold larger. Thus, *MyD88* kd reduced the ZOI by 44.5% compared with the ds*GFP* control. Similarly, injection of ds*MyD88* reduced the fold induction of antimicrobial activity after the *E. coli* (43.6%) and *S. aureus* (46.3%) challenge compared with the ds*GFP*-injected mosquitoes challenged with the same bacterial species. Therefore, silencing *MyD88* negatively impacted the antimicrobial activity in the hemolymph after the bacterial challenge, phenocopying *REL1*-silencing.

In its unphosphorylated state, the inhibitor of (I)κB CACT is bound to REL1, preventing it from entering the nucleus. Thus, removal of CACT leads to a constitutive activation of the Toll pathway (Frolet et al., 2006; Riehle et al., 2008; Garver et al., 2009) (**Figure 3A**). To determine the constitutive immune activation and to explore the temporal variation of antimicrobial activity after injection of ds*CACT*, we collected hemolymph at 24, 48, 60, and 72 h after dsRNA injections. The antimicrobial activity of hemolymph was significantly increased in ds*CACT*-injected mosquitoes compared with UC and ds*GFP*-injected controls at every timepoint (**Figure 3D**; one-way ANOVA, *df* = 11, *F* = 102.4, *P* < 0.0001, Bonferroni’s post-test, *P* < 0.0001). At the 24-h timepoint, hemolymph from ds*CACT*-injected mosquitoes produced a twofold larger ZOI than the hemolymph UC. The antimicrobial activity after ds*CACT* injection further increased at 48 h, with a ZOI 6.6-fold larger than the UC and 2.2-fold larger than at the 24-h timepoint. The activity at 60 h after ds*CACT* injection was reduced by 39.8% compared with the 48-h timepoint but remained high overall, with a ZOI 4.8-fold larger than the UC at the same timepoint. At 72 h, the antimicrobial activity was reduced to levels comparable with the 24-h timepoint, producing a ZOI 2.3-fold larger than the hemolymph of UC but 60.8% and 34.9% smaller than the hemolymph of ds*CACT*-injected mosquitoes at 48 and 60 h, respectively. Overall, these data confirmed that constitutive immune activation through ds*CACT* injection can be detected through ZOI assays. The antimicrobial activity peaked at 48 h after ds*CACT* injection, after which the activity plateaued but remained elevated for at least an additional 24 h.

**Figure 4:**
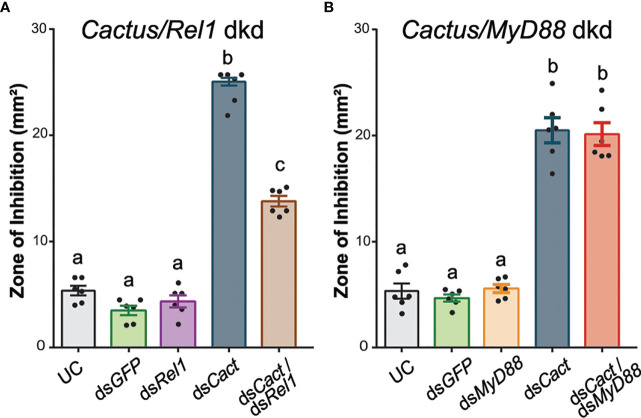
Ds*REL1* injection partially reverses the antimicrobial activity induced by *CACT* kd. **(A)** Zone of inhibition (ZOI) produced by the hemolymph of mosquitoes injected with ds*REL1*/ds*GFP*, ds*CACT*/ds*GFP*, and ds*CACT*/ds*REL1.* Uninjected and ds*GFP-*injected mosquitoes acted as negative controls. **(B)** ZOI produced by the hemolymph of mosquitoes injected with ds*MyD88*/ds*GFP*, ds*CACT*/ds*GFP*, and ds*CACT*/ds*MyD88.* Uninjected (UC) and ds*GFP-*injected mosquitoes acted as negative controls. Data are shown as means ±1 standard error (*n* = 6). One-way ANOVA, followed by Bonferroni’s multiple-comparison post-test, was performed to calculate the statistical significance (*P* < 0.05). Means with the same letter are not significantly different.

To confirm that the elevated antimicrobial activity observed after ds*CACT* injection can be explained by canonical Toll signaling (**Figure 3A**), we tested the effect of co-silencing *REL1* and *CACTUS.* The kd efficiency of *CACT/REL1* was measured 48 h after dsRNA injections (**Supplementary Table S3**). The antimicrobial activity of hemolymph was significantly reduced in ds*REL1/*ds*CACT-*injected mosquitoes compared with the ds*CACT*-injected controls (**Figure 4A**; one-way ANOVA, *df* = 4, *F* = 382.2, *P* < 0.0001, Bonferroni’s post-test, *P* < 0.0001). Hemolymph antimicrobial activity did not increase by doubling the amount of injected ds*GFP* nor by injecting ds*RELl1*/ds*GFP* compared with the UC (Bonferroni’s post-test, *P* > 0.999). Similar to the previously observed increases in ZOI in the single-knockdown assays (**Figure 3A**), the antimicrobial activity was increased 6.1-fold in hemolymph from ds*CACT*/ds*GFP*-injected mosquitoes compared with the UC. Co-injection of ds*CACT* and ds*REL1* reduced the ZOI by 45% when compared with ds*CACT*/ds*GFP*-injected mosquitoes, demonstrating that *REL1* is required for the antimicrobial activity induced by ds*CACT* injection.

In contrast, the antimicrobial activity in hemolymph collected from mosquitoes co-injected with ds*MyD88* and ds*CACT* was comparable with that of hemolymph from ds*CACT*/ds*GFP*-injected mosquitoes (**Figure 4B**; one-way ANOVA, *df* = 4, *F* = 103.0, *P* < 0.0001, Bonferroni’s post-test, *P* > 0.999). The kd efficiency of *CACT/MyD88* was measured 48 h after dsRNA injections (**Supplementary Table S3**). The ZOI produced by hemolymph collected from ds*MyD88*/ds*GFP*-injected mosquitoes was not statistically significantly different from that of UC mosquitoes (Bonferroni’s post-test, *P* > 0.999). The antimicrobial activity after ds*CACT*/ds*GFP* injections was increased compared with that of UC hemolymph and not statistically significantly different from that of ds*CACT*/ds*MyD88*-injected mosquitoes. Thus, ds*MyD88* injection did not affect the antimicrobial activity induced by *dsCACT* injection.

These data altogether demonstrate that ZOI assays provide a convenient means to evaluate the contribution of the Toll pathway to the antimicrobial activity in *A. gambiae* hemolymph. Our data demonstrate that REL1 and MyD88 are required for the antimicrobial activity against *M. luteus* and that this activity is inhibited by CACT. In addition, the dynamic range of the assay is sufficient to perform epistatic analyses and confirm that MyD88 is upstream of CACT in the Toll pathway in *A. gambiae*.

### Injection induces a priming response

**Figure 5:**
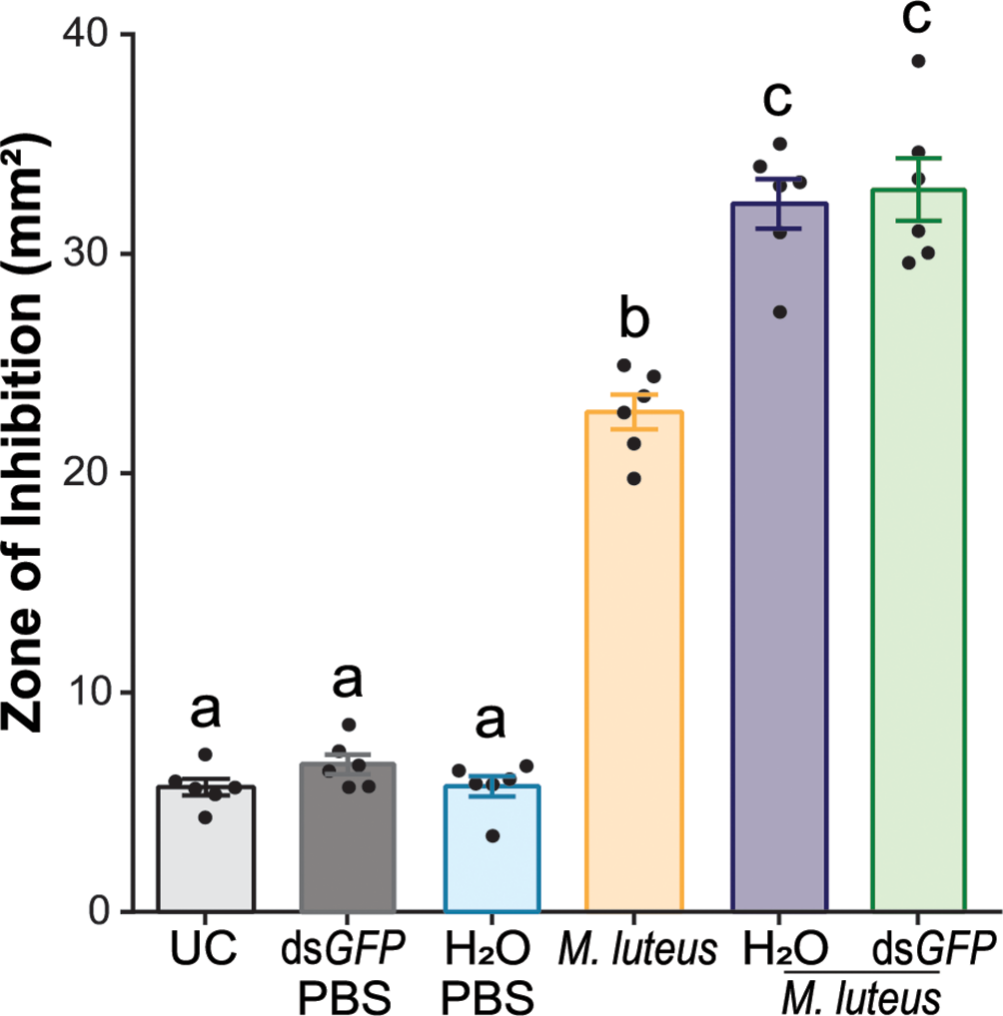
Intrathoracic injection enhances the efficacy of bacterial growth inhibition after bacterial challenge. The graph shows the zone of inhibition (ZOI) produced by the hemolymph of mosquitoes injected with sterile deionized water (H_2_O) or ds*GFP*, followed by phosphate-buffered saline injection, *M. luteus* challenge only, and H_2_O and ds*GFP* followed by *M. luteus* challenge. The mosquitoes were challenged 48 h after dsRNA or H_2_O injection. The hemolymph of uninjected mosquitoes acted as negative controls. Data are shown as means ±1 standard error (*n* = 6). One-way ANOVA, followed by Bonferroni’s multiple-comparison post-test, was performed to calculate the statistical significance (*P* < 0.05). Means with the same letter are not significantly different.

Across all the experiments that required immune activation *via* bacterial challenge, we consistently observed a larger ZOI when ds*GFP* was injected prior to challenge, suggesting a priming response. To determine whether the injury itself or the presence of dsRNA was responsible for this effect, we injected mosquitoes with either ds*GFP* resuspended in sterile deionized water (H_2_O) or H_2_O only, followed by *M. luteus* challenge (**Figure 5**). The antimicrobial activity of the hemolymph of UC mosquitoes was low and did not increase in the hemolymph of H_2_O- or ds*GFP*-injected mosquitoes that were injected 48 h later with PBS (one-way ANOVA, *df* = 5, *F* = 235.3, *P* < 0.0001, Bonferroni’s post-test, *P* > 0.999). As demonstrated previously (**Figures 1B, C**), *M. luteus* challenge significantly increased the antimicrobial activity (threefold) in hemolymph compared with the UC (Bonferroni’s post-test, *P* < 0.0001). Interestingly, the antimicrobial activity in the hemolymph of H_2_O- and ds*GFP*-injected mosquitoes subsequently challenged with *M. luteus* was significantly higher compared with that of mosquitoes only challenged with *M. luteus* (41.7% and 44.5%, Bonferroni’s post-tests, *P* < 0.0001 for both). The antimicrobial activity was comparable between the hemolymph of H_2_O- and ds*GFP*-injected mosquitoes subsequently challenged with *M. luteus* (Bonferroni’s post-test, *P* > 0.999). These results demonstrate that antimicrobial activity induction in mosquito hemolymph is primed by injection. This effect, however, is not exacerbated by the injection of dsRNA molecules.

## Discussion

### Using ZOI assays to quantify humoral antimicrobial activity in *A. gambiae*

In this study, we propose a new application of ZOI assays based on reverse genetics approach, which aims to identify the contribution of specific immune factors to the antimicrobial activity of *A. gambiae* hemolymph. Previous assays to identify factors that contribute to antimicrobial immunity in *A. gambiae* have largely relied on *in vivo* assays, including mosquito survival after injection of live bacteria (Blandin et al., 2002; Gupta et al., 2009; Coggins et al., 2012), observations of phagocytosis *in vivo* (Moita et al., 2005), and measurements of bacterial proliferation after challenge (Schnitger et al., 2007; Yassine et al., 2012). However, survival and bacterial proliferation are dependent on both cellular and humoral immune responses (Dimopoulos et al., 1997; Christophides et al., 2004; Levashina, 2004; Hillyer and Strand, 2014), and thus these assays are not able to distinguish between factors contributing to phagocytosis, humoral immunity, or both. ZOI assays specifically measure the humoral arm of the antimicrobial immune response and thus provide a much-needed tool for the analysis of immunity in *A. gambiae*.

The humoral antimicrobial activity that we detected in the ZOI assays was not specific to bacterial species that were used in the challenges. Indeed all challenges that we employed—lyophilized *M. luteus*, live *E. coli*, and *S. aureus*—induced antimicrobial activity against *M. luteus*, further supporting the literature that different microbial challenges induce an overlapping repertoire of humoral innate immune responses that broadly defend against an array of bacterial species (Coggins et al., 2012; Rhodes et al., 2018).

Despite our efforts, we could, however, not establish similar ZOI assays against other microbial species, including *E. coli*, *S. aureus*, and *S. cerevisiae*. This parallels the findings by League et al. (2017), where the hemolymph of *A. gambiae* larvae also was only able to limit the growth of *M. luteus* and not *E. coli*. Similarly, the hemolymph of adult *Tenebrio molitor* did not limit the growth of *E. coli* but only that of *Arthrobacter globiformis*, and *M. luteus* was not tested in this study (Haine et al., 2008). The authors speculated that the replication rate of the microbe tested may be responsible for these observations, suggesting that fast-replicating species in this *in vitro* assay outpace the antimicrobial activity of hemolymph, thus precluding the measurements of ZOI (Haine et al., 2008; League et al., 2017). However, when we reduced the incubation temperature to slow down bacterial replication, we still could not observe a ZOI. Another potential explanation for the lack of ZOIs against *E. coli* in *A. gambiae* hemolymph may be the more limited sensitivity of the ZOI assay when using microbial species other than *M. luteus.* Such limited sensitivity would disproportionately affect ZOI assays using insect species with a small individual hemolymph volume. Indeed several studies that use hemolymph from larger insect species, including *Manduca sexta*, *Hyalophora cecropia*, *Antheraea mylitta*, *Reticulitermes flavipes*, and *Diatraea saccharalis*, have indeed detected ZOIs in plates seeded with *E. coli*, *Staphylococcus aureus*, and *Klebsiella pneumoniae* (Hultmark et al., 1983; Booth et al., 2015; Rocha et al., 2016; Zeng et al., 2016; Chowdhury et al., 2021). Given the size of mosquitoes and the volume of hemolymph that we collected and used for the assay, it is possible that using higher volumes of hemolymph would show bacterial growth inhibition in plates. **Supplementary Table S4** summarizes the uses and limitations of ZOI assays compared with other assays commonly used to assess humoral antimicrobial activity in mosquitoes. In the future, the sensitivity of ZOI assays using *A. gambiae* hemolymph may be further increased by adding commercial chicken egg white lysozyme into the *E. coli*-seeded agarose, as demonstrated by Chalk et al. (1994).

### AMPs are responsible for the antimicrobial activity in mosquito hemolymph

Our data show that performing gene knockdown by dsRNA injection prior to the ZOI assays can be used to identify the major factors underlying the antimicrobial activity in *A. gambiae*. We tested the contribution of AMP genes to antimicrobial activity and showed that, in the hemolymph of adult female *A. gambiae*, *DEF1* is largely responsible for the antimicrobial activity against *M. luteus*. This *ex vivo* observation of the antimicrobial activity of DEF1 using ZOI assays is consistent with *in vitro* results of the antimicrobial activity spectrum of recombinant DEF1. Most of the Gram-positive bacterial species tested were sensitive in a range of concentrations from 0.1 to 0.75 μM of recombinant DEF1. At 1 µM, DEF1 exhibited a strong bactericidal effect on *M. luteus*, killing almost all bacteria within 60 s (Vizioli et al., 2001b). In addition, our data corroborate the *DEF1* kd findings by Blandin et al. (2002) that showed that this AMP is required for the mosquito antimicrobial defense against Gram-positive bacteria in female *A. gambiae*. In this study, *DEF1* depletion rendered the mosquitoes more susceptible to *M. luteus* (20% lethality) and highly susceptible to *S. aureus* (80% lethality) infections.

In addition to *DEF1*, we also found that *CEC1* contributed to antimicrobial activity in *A. gambiae* hemolymph, albeit to a lesser extent than *DEF1*. CEC1 is produced in bacteria-inoculated adults, where mature CEC1 can be found in two forms—C-terminally amidated and glycine-extended (non-amidated) forms—showing minimal differences in antimicrobial spectrum (Vizioli et al., 2000). Both forms of CEC1 show a strong bactericidal effect on *M. luteus*, with 0.5–1 and 1–2.5 uM minimal inhibitory concentrations (MIC) for the amidated and glycine-extended forms, respectively (Vizioli et al., 2000). The *in vitro* bactericidal effects of DEF1 and CEC1 on *M. luteus* are similar and thus do not explain the smaller ZOIs that we observed after *CEC1* kd. The underlying cause of this difference is currently unclear. It is possible that this difference can be attributed to differences in kd levels, which could be addressed by producing CRISPR-knockout strains. An alternative and currently equal parsimonious explanation is that the DEF1 and CEC1 peptide levels differ in the hemolymph of adult female *A. gambiae* after *M. luteus* challenge.

Overall, our results provide further evidence that not only AMPs are required for antimicrobial activity in *A. gambiae* hemolymph but also the induction of antimicrobial activity against *M. luteus* after bacterial challenge is largely explained by *DEF1.*

### Humoral antimicrobial activity in *A. gambiae* hemolymph is regulated by the Toll pathway

Our results confirm previous reports that the Toll pathway is the major regulator of antimicrobial activity in the mosquito hemolymph through several lines of evidence (Christophides et al., 2002). First, humoral antimicrobial activity was reduced in the hemolymph of bacterially challenged adult female insects injected with ds*REL1*. This confirms the results in previous studies in *A. gambiae* that identified AMPs, including *DEF1*, as *REL1-*regulated immune genes (Frolet et al., 2006; Luna et al., 2006). Additionally, our data show that the antimicrobial activity in the mosquito hemolymph increased after silencing *CACT*. This increase is explained by the gene overexpression of several AMPs, including *CEC1*, *DEF1*, *Gambicin 1* (*GAM1*), and *CEC3* induced by the silencing of *CACT* in *A. gambiae* (Garver et al., 2009). This increase in antimicrobial activity is partially reversed in ds*CACT/*ds*REL1*-injected mosquitoes, further supporting that the increased activation of humoral antimicrobial activity by *CACT* depletion is dependent on the downstream NF-kB transcription factor REL1 as shown previously at the transcriptional level (Garver et al., 2009). Our data parallel previous findings on antimicrobial activity against the fungal entomopathogen *Beauveria bassiana* as well as on antiparasitic activity against the rodent malaria parasite *Plasmodium berghei*, both of which can be boosted by *CACT* kd in a *REL1*-dependent manner (Frolet et al., 2006; Rhodes et al., 2018).

Furthermore, to our knowledge, we provide the first evidence of the death-domain adapter protein MyD88 to be required for humoral antimicrobial immunity in *A. gambiae*. In *D. melanogaster*, MyD88 is a component of the Toll pathway, acting upstream of CACT (Horng and Medzhitov, 2001; Tauszig-Delamasure et al., 2002). *MyD88* in *A. gambiae* was first identified as a 1:1 ortholog during the initial genome annotation of the *A. gambiae* PEST strain (Christophides et al., 2002). An injection of ds*MyD88* reduced the antimicrobial activity of *A. gambiae* hemolymph after bacterial challenge, phenocopying the effect of *dsREL1* injection. However, injection of ds*MyD88* did not impact the ds*CACT* phenotype in ds*CACT/*ds*MyD88*-injected mosquitoes, supporting a placement of MyD88 in the Toll pathway upstream of CACT.

These data together provide strong evidence that the Toll pathway directly regulates humoral anti-bacterial activity in the hemolymph of adult *A. gambiae*. Importantly, the data also show that the dynamic range of the *A. gambiae* hemolymph ZOI assays is sufficient to perform epistasis-like analyses by dsRNA injections. The latter will be invaluable to decipher regulatory components of the Toll pathway upstream of its receptor.

### Bacteria challenge induces the highest antimicrobial activity 24 h after bacterial injection

While not the focus of our study, measurement of antimicrobial activity after bacterial challenge and ds*CACT* injection revealed the temporal nature of the humoral immune responses to bacterial challenge in adult female *A. gambiae*. In our study, hemolymph antimicrobial activity, upon bacterial challenge, reached maximal levels between 24 and 48 h and peaked at 48 h after ds*CACT*-injection. Several studies previously described the rapid induction of RNA levels of *DEF1*, *CEC1*, and *GAM1* in adult *A. gambiae* (Dimopoulos et al., 1997; Vizioli et al., 2000; Vizioli et al., 2001a; Vizioli et al., 2001b). In addition, previous reports from other insects such as *Zophobas atratus* and *Bombus terrestris* show that the time at which hemolymph antimicrobial activity peaks after a challenge varies between 24 and 48 h (Bulet et al., 1991; Korner and Schmid-Hempel, 2004).

Furthermore, we observed a persistence of elevated antimicrobial activity for at least up to 72 h. Antimicrobial activity lasting at least 5 days has been observed in the butterfly *Pieris brassicae* and the wax moth *Galleria mellonella* (Jarosz, 1993) and up to 44 days after challenge in the dragonfly *Aeschna cyanea* (Bulet et al., 1992). In *A. gambiae*, *E. coli* challenge stably induces the expression of *DEF1* mRNA over a period of at least 8–12 days (Gorman and Paskewitz, 2000; Blandin et al., 2002), whereas the gene expression of *DEFA*, the 1:1 ortholog of *DEF1* in the mosquito *Aedes aegypti*, remains abundant for at least 21 days (Lowenberger et al., 1999). However, whether this elevated gene expression is translated to sustained elevated peptide levels is currently unknown, especially because data on *A. aegypti* show that the expression of *DEFA* is regulated post-transcriptionally and that elevated mRNA levels do not translate to elevated peptide levels (Bartholomay et al., 2004).

Future studies will have to determine whether the persistence of antimicrobial activity in *A. gambiae* after *M. luteus* challenge is due to continued elevated expression levels and/or the persistence of certain antimicrobial peptides.

### Injection is a stressor that triggers antimicrobial activity

The lasting effect of antimicrobial activity in the hemolymph suggests that, in secondary challenges, initial AMP levels may be elevated compared with those in hemolymph from naïve mosquitoes. Our results show that increased antimicrobial activity upon a secondary challenge is a consequence of previously activated defenses. This phenomenon of immune memory, reliant on the innate immune system, is referred to as immune priming or trained immunity (Boman et al., 1972; Moret and Schmid-Hempel, 2001; Apidianakis et al., 2005; Schmid-Hempel, 2005; Pham et al., 2007; Sun et al., 2009; Netea et al., 2011; Christofi and Apidianakis, 2013).

Immune priming in *A. gambiae* has been described in response to injury, bacterial challenge, and malaria parasite infection (Rodrigues et al., 2010; Powers et al., 2020). Injury or bacterial challenge leads, in secondary bacterial infections, to a decreased bacterial load and increased survival in adult female *A. gambiae* (Powers et al., 2020). Similarly, malaria parasite infection loads were decreased if mosquitoes were previously infected with the same parasite species (Rodrigues et al., 2010). While the molecular mechanisms underlying the priming effect of bacteria or injury is unknown, priming by parasite infection is mediated by hemocyte differentiation (Ramirez et al., 2014).

Our data suggest that priming by injury is, at least in part, based on humoral factors, as the ZOI assays are cell-free. While we cannot exclude that injury is accompanied by the introduction of a small number of environmental bacteria from the mosquito surface or environmental bacteria present in dH_2_O or ds*GFP*, the priming effect that we observed is not explained simply by the continued elevation of AMP levels, as injury through either injection of water or dsRNA did not increase the antimicrobial activity upon the initial injury itself or subsequent secondary injury. Increased antimicrobial activity was instead only observed after secondary bacterial challenge following the initial injury. Future studies will have to determine the molecular underpinnings of this injury-based priming and will have to rely on reverse genetics methodology that does not require injury.

In conclusion, the novel use of ZOI assays that we propose in this study represents an *ex vivo* low-biosafety-level, medium-throughput assay that can be used to provide information on the regulation of antimicrobial activity in response to microbial infections in mosquitoes. Modifications to this assay, such as the use of a device that will allow us to produce uniform and equally spaced wells at the same time, will further streamline the generation of plates. Alternatively, using a MIC assay based on broth dilution methods may allow us to measure the differences in antimicrobial activity with bacterial species besides *M. luteus*. Importantly, beyond differences in antimicrobial activity after silencing the genes involved in the intracellular cascade of Toll pathway, this assay can be extended to determine the extracellular regulatory elements of antimicrobial activity in *A. gambiae* hemolymph.

## Data availability statement

The original contributions presented in the study are included in the article/**Supplementary Material**. Further inquiries can be directed to the corresponding authors.

## Author contributions

BM and KM conceived the original idea and designed the experiments. BM performed the experiments and analyzed the data. KM supervised the project. BM and KM drafted the manuscript and contributed to the final version of the manuscript. All authors contributed to the article and approved the submitted version.
