# Supplementary material for: A zone-of-inhibition assay to screen for humoral antimicrobial activity in mosquito hemolymph
Source: Front Cell Infect Microbiol. 2023 Jan 26;13:891577. doi: 10.3389/fcimb.2023.891577 (PMC9908765; doi:10.3389/fcimb.2023.891577)
Supplement: Supplementary file 1 [file Image_1.pdf]

Figure S1.

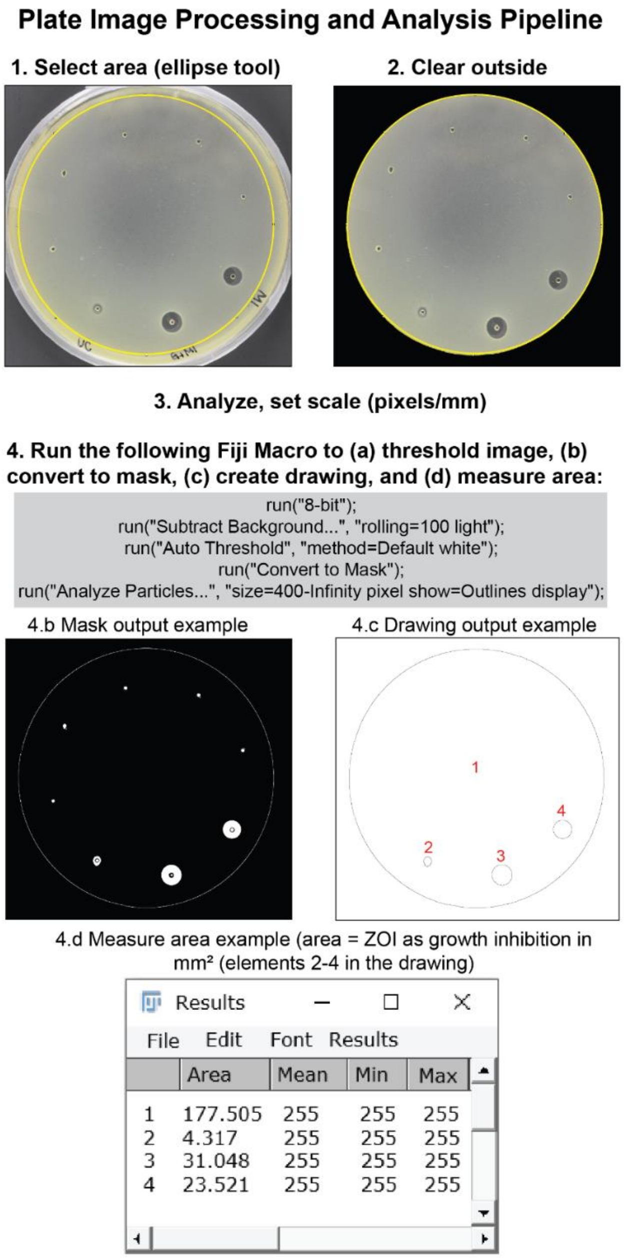

Figure S1. Overview of the bacterial plate imaging and ZOI data analysis pipeline. Plates were imaged at 16.1 pixels per mm using the Azure 300 imaging system and processed in Fiji is Image J. The area where the wells loaded with hemolymph is located is selected with the ellipse tool (1), excluding the labels and edges of the plate by selecting the “Clear outside” option in the “Edit” menu (2). The scale used for the images can be input by selecting the “Set scale” option in the “Analyze” menu (3). Once the scale is defined, the provided macro is run by selecting “Run”> “Macro” in the menu “Plugins”. The macro establishes a threshold for the image (4a), converts it into a mask (4b), and creates a drawing (4c) to measure the area of growth inhibition per well (4d).
