# Supplementary material for: A zone-of-inhibition assay to screen for humoral antimicrobial activity in mosquito hemolymph
Source: Front Cell Infect Microbiol. 2023 Jan 26;13:891577. doi: 10.3389/fcimb.2023.891577 (PMC9908765; doi:10.3389/fcimb.2023.891577)
Supplement: Supplementary file 2 [file Image_2.pdf]

Figure S2.

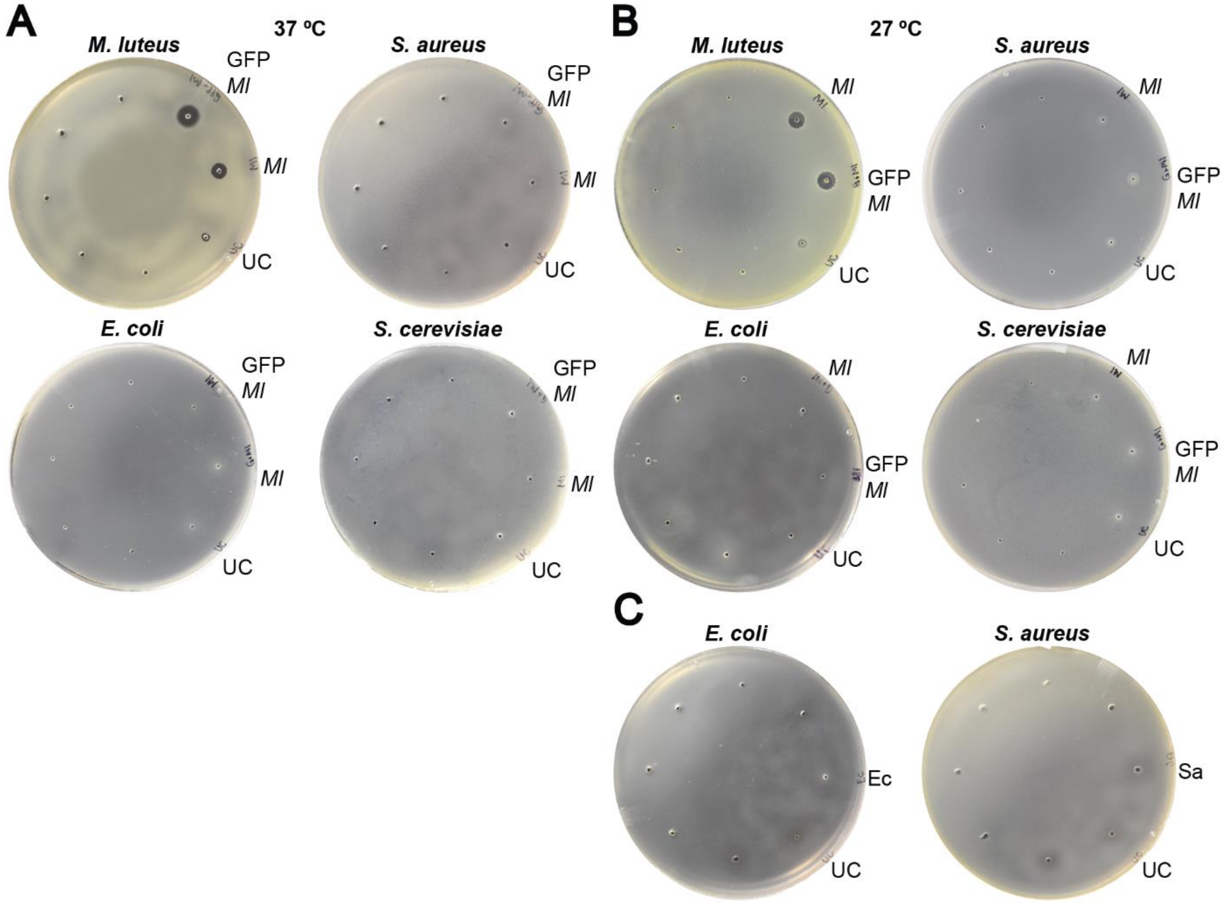

Figure S2. *Anopheles* hemolymph only inhibits growth of *M. luteus*, not *E. coli*, *S. aureus*, or *S. cerevisiae*. Plates seeded with *M. luteus* (top left), *S. aureus* (top right), *E. coli* (bottom left), and *E. coli* (bottom right) incubated at (**A**) 37˚C, and (**B**) 27˚C. Hemolymph samples from mosquitoes that were unchallenged (UC), *M. luteus*-challenged, and ds*GFP*-injected prior to *M. luteus* challenge were tested on these plates. (**C**) Plates seeded with *S. aureus* (right), *E. coli* (left), incubated at 27˚C. Hemolymph samples from mosquitoes that were unchallenged (UC), *S. aureus* and *E. coli*-challenged were tested on their correspondent plates.
