# Supplementary material for: A zone-of-inhibition assay to screen for humoral antimicrobial activity in mosquito hemolymph
Source: Front Cell Infect Microbiol. 2023 Jan 26;13:891577. doi: 10.3389/fcimb.2023.891577 (PMC9908765; doi:10.3389/fcimb.2023.891577)
Supplement: Supplementary file 3 [file Image_3.pdf]

Figure S3.

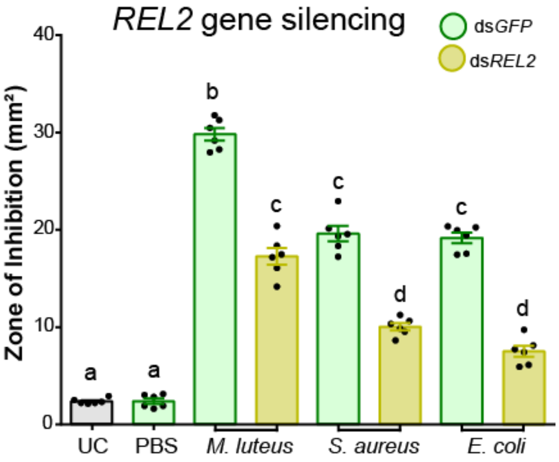

Figure S3. *REL2* gene silencing reduces antimicrobial activity similarly to *REL1* kd. ZOI produced by the hemolymph of ds*REL2*-injected mosquitoes. Mosquitoes were challenged 48 h after dsRNA injection with *E. coli*, *S. aureus*, and *M. luteus*, respectively. Hemolymph was collected 24 h after challenge. Mosquitoes that were uninjected (UC) and injected with ds*GFP* prior to PBS acted as negative controls, whereas ds*GFP* injection prior to *E. coli*, *S. aureus*, and *M. luteus* challenges acted as the corresponding positive controls.
