# Supplementary material for: A zone-of-inhibition assay to screen for humoral antimicrobial activity in mosquito hemolymph
Source: Front Cell Infect Microbiol. 2023 Jan 26;13:891577. doi: 10.3389/fcimb.2023.891577 (PMC9908765; doi:10.3389/fcimb.2023.891577)
Supplement: Supplementary file 4 [file Table_1.pdf]

Supplementary Materials

Table S1. dsRNA Primer sequences

Primer Function	Primer Name	Primer Sequences (5'-3')	Primer design
1st round template amplification	dsGFP_F	TAATACGACTCACTATAGGGCGATGC	An et al., 2011
	dsGFP_R	TAATACGACTCACTATAGGGCGGACT	
	dsCEC1_F	TAATACGACTCACTATAGGGATCAACCCAGAGACCAACCA	This study
	dsCEC1_R	TAATACGACTCACTATAGGGATGTTAGCAGAGCCGTCGTC	
	dsDEF1_F	TAATACGACTCACTATAGGGCTGTGCCTTCCTAGAGCAT	Blandin et al., 2002
	dsDEF1_R	TAATACGACTCACTATAGGGCACACCCTCTTCCCAGGAT	
	dsCACT_F	TAATACGACTCACTATAGGGTAACACTGCGCTTCATTTGG	Rhodes et al., 2018
	dsCACT_R	TAATACGACTCACTATAGGGGCCCTTTTCAATGCTGATGT	
	dsREL1_F	TAATACGACTCACTATAGGGGGGCAGTGGTCGTTGTGT	Rhodes et al., 2018
	dsREL1_R	TAATACGACTCACTATAGGGGCACTGAATGCCCAAATTGT	
	dsREL2_F	TAATACGACTCACTATAGGGCACCTATGCCAGTGCGCTT	This study
	dsREL2_R	TAATACGACTCACTATAGGGCGCTACTGGAAAGGTTCAGG	
	dsMyD88_F	TAATACGACTCACTATAGGGAGCAAACCGGTGGAATTGAT	This study
	dsMyD88_R	TAATACGACTCACTATAGGGTGCTCCCGTTTAGTTTGTCC	
2nd round template amplification	T7	TAATACGACTCACTATAGGG	Zhang et al., 2016

Added sequence for T7 promoter is underlined
