# Supplementary material for: A zone-of-inhibition assay to screen for humoral antimicrobial activity in mosquito hemolymph
Source: Front Cell Infect Microbiol. 2023 Jan 26;13:891577. doi: 10.3389/fcimb.2023.891577 (PMC9908765; doi:10.3389/fcimb.2023.891577)
Supplement: Supplementary file 5 [file Table_2.pdf]

Table S2. RT-qPCR Primer sequences and efficiencies

Primer Name	Primer Sequences (5'-3')	Primer design	Slope ± SE	R^2	*P*	Efficiency	
qS7_F<br>qS7_R	GTGCGCGAGTTGGAGAAGA<br>ATCGGTTTGGGCAGAATGC	Michel et al., 2005	-3.107 ± 0.080	0.987	<0.0001	2.10	109.8%
qCEC1_F<br>qCEC1_R	GCAGTCAGACGGAAG<br>CTTGAACACTCGCTTG	This study	-3.161 ± 0.081	0.987	<0.0001	2.07	107.2%
qDEF1_F<br>qDEF1_R	TTCCGTAGCAACCCCGAG<br>GCTGCGAAACGAATTAAGCC	This study	-3.174 ± 0.097	0.982	<0.0001	2.06	106.6%
qCACT_F<br>qCACT_R	AATCTGGGCCTGATGGACA<br>ACTGCCAGGTGCAGTTGAGT	Rhodes et al., 2018	-3.238 ± 0.105	0.980	<0.0001	2.04	103.6%
qREL1_F<br>qREL1_R	TCAACAGATGCCAAAAGAGGAAAT<br>CTGGTTGGAGGGATTGTG	Rhodes et al., 2018	-3.181 ± 0.066	0.993	<0.0001	2.06	106.2%
qMyD88_F<br>qMyD88_R	ATTACCCAGCCCGAAGAAGG<br>GGCTTTATCACTGGCGGAAG	This study	-3.177 ± 0.149	0.972	<0.0001	2.06	106.4%
qREL2_F<br>qREL2_R	CGGGCAGAGGGAAGCAT<br>AGGCCCGCTCACCGTT	This study	-3.083 ± 0.103	0.979	<0.0001	2.11	110.9%
