# Supplementary material for: A zone-of-inhibition assay to screen for humoral antimicrobial activity in mosquito hemolymph
Source: Front Cell Infect Microbiol. 2023 Jan 26;13:891577. doi: 10.3389/fcimb.2023.891577 (PMC9908765; doi:10.3389/fcimb.2023.891577)
Supplement: Supplementary file 6 [file Table_3.pdf]

Table S3. Gene knockdown efficiency

Target gene	Treatment	Relative expression ratio (*R*)* ± SE
CEC1	ds*GFP*	1 ± 0.0
	ds*CEC1*	0.499 ± 0.016
DEF1	ds*GFP*	1 ± 0.0
	ds*DEF1*	0.096 ± 0.029
CACT	ds*GFP*/ds*GFP*	1 ± 0.0
	ds*CACT*/ds*GFP*	0.509 ± 0.022
	ds*CACT*/ds*REL1*	0.482 ± 0.025
	ds*CACT*/ds*MyD88*	0.584 ± 0.024
REL1	ds*GFP*/ds*GFP*	1 ± 0.0
	ds*REL1*/ds*GFP*	0.428 ± 0.038
	ds*CACT*/ds*REL1*	0.689 ± 0.141
MyD88	ds*GFP*/ds*GFP*	1 ± 0.0
	ds*MyD88*/ds*GFP*	0.379 ± 0.024
	ds*CACT*/ds*MyD88*	0.397 ± 0.038
REL2	ds*GFP*	1 ± 0.0
	ds*REL2*	0.109 ± 0.019

*RT-qPCR was used to measure the relative expression levels with ribosomal protein *S7* as the internal reference and ds*GFP*-treated samples as the calibrator condition. *R* calculated using the Pfaffl (2001) mathematical model for relative quantification in RT-qPCR.
