# Supplementary material for: A zone-of-inhibition assay to screen for humoral antimicrobial activity in mosquito hemolymph
Source: Front Cell Infect Microbiol. 2023 Jan 26;13:891577. doi: 10.3389/fcimb.2023.891577 (PMC9908765; doi:10.3389/fcimb.2023.891577)
Supplement: Supplementary file 7 [file Table_4.pdf]

Table S4. Uses and limitations of ZOI assays.

Assays to measure humoral antimicrobial activity in mosquitoes	Advantages	Limitations
Mosquito survival after bacterial challenge	Endpoint measure that does not require further manipulation after challenge<br>*In vivo*	Does not discriminate between impact on resistance vs. tolerance to infection<br>Does not discriminate between cellular and humoral immunity<br>Indirect measure of antibacterial activity<br>Requires large number of mosquitoes per assay
Bacterial proliferation	Discriminates between impact on infection resistance vs. tolerance to infection<br>Smaller number of mosquitoes as compared to survival curves and ZOI assays<br>*Ex vivo*	Does not discriminate between cellular and humoral immunity<br>Indirect measure of antibacterial activity<br>Requires further manipulation after challenge, including extraction, serial dilutions or expensive equipment, enumeration of colony forming units<br>Requires a bacterial marker to discriminate between infectious agent and resident microbiota
Zone of Inhibition (ZOI)	Direct measure of hemolymph impact on bacterial growth<br>Assesses only humoral antibacterial activity, excluding melanization and opsonization<br>*Ex vivo*	Currently limited to measure impact on *Micrococcus luteus* growth<br>Requires large number of mosquitoes per assay<br>Requires further manipulation after challenge, including hemolymph extraction, plate preparation, and quantification of ZOIs
Quantification of antimicrobial peptide gene (AMP) expression	Smaller number of mosquitoes as compared to survival curves and ZOI assays<br>Indirect measure of infection impact on resistance<br>*Ex vivo*<br>High reproducibility	The relative contribution of signaling pathways that contribute to the expression of individual AMPs is largely unclear<br>Does not assess impact on antimicrobial immunity<br>Unclear how changes in gene expression affect AMP protein levels in and antimicrobial activity of mosquito hemolymph<br>Requires further manipulation after challenge, including RNA extraction, individual primer pairs for each AMP gene, and significant time investment to set up each assay<br>Expensive equipment and reagents, although cost has declined over recent years, and smaller, field-deployable equipment is now available
Quantification of AMPs	Smaller number of mosquitoes as compared to survival curves and ZOI assays<br>Indirect measure of infection impact on resistance<br>*Ex vivo*	Does not assess impact on antimicrobial immunity<br>Unclear how changes in AMP concentration affect the antimicrobial activity of mosquito hemolymph<br>Requires further manipulation after challenge, including hemolymph extraction, separation of plasma proteins, mass spectrometry sample preparation and analysis, and significant time investment to set up each assay<br>Expensive equipment and skilled downstream analyses
